# Joint modeling of multivariate longitudinal data and the dropout process in a competing risk setting: application to ICU data

**DOI:** 10.1186/1471-2288-10-69

**Published:** 2010-07-29

**Authors:** Emmanuelle Deslandes, Sylvie Chevret

**Affiliations:** 1 Département de Biostatistique et Informatique Médicale, Hôpital Saint-Louis, AP-HP, Paris, France; 2 Université Paris 7 - Denis Diderot, Paris, France; 3 Inserm, UMRS 717, Paris, France

## Abstract

**Background:**

Joint modeling of longitudinal and survival data has been increasingly considered in clinical trials, notably in cancer and AIDS. In critically ill patients admitted to an intensive care unit (ICU), such models also appear to be of interest in the investigation of the effect of treatment on severity scores due to the likely association between the longitudinal score and the dropout process, either caused by deaths or live discharges from the ICU. However, in this competing risk setting, only cause-specific hazard sub-models for the multiple failure types data have been used.

**Methods:**

We propose a joint model that consists of a linear mixed effects submodel for the longitudinal outcome, and a proportional subdistribution hazards submodel for the competing risks survival data, linked together by latent random effects. We use Markov chain Monte Carlo technique of Gibbs sampling to estimate the joint posterior distribution of the unknown parameters of the model. The proposed method is studied and compared to joint model with cause-specific hazards submodel in simulations and applied to a data set that consisted of repeated measurements of severity score and time of discharge and death for 1,401 ICU patients.

**Results:**

Time by treatment interaction was observed on the evolution of the mean SOFA score when ignoring potentially informative dropouts due to ICU deaths and live discharges from the ICU. In contrast, this was no longer significant when modeling the cause-specific hazards of informative dropouts. Such a time by treatment interaction persisted together with an evidence of treatment effect on the hazard of death when modeling dropout processes through the use of the Fine and Gray model for sub-distribution hazards.

**Conclusions:**

In the joint modeling of competing risks with longitudinal response, differences in the handling of competing risk outcomes appear to translate into the estimated difference in treatment effect on the longitudinal outcome. Such a modeling strategy should be carefully defined prior to analysis.

## Background

When evaluating the efficacy of a new drug through randomized clinical trials (RCT) in critically ill patients, the primary endpoint of interest is usually death from any cause within some fixed period, generally 28 or 90 days after randomization. However, to better investigate the effect of treatment, one is often interested in evaluating how a biomarker of interest changes over time and how this change may be correlated with the treatment under study; this defines secondary endpoints of interest.

In critically ill patients, the measure of treatment effectiveness is based on the severity of the illness and degree of organ failure, determined using severity scores such as the APACHE (acute physiology and chronic health evaluation) II score [1] or the Glasgow coma score [2] and the SOFA (sequential organ failure assessment) score [3], respectively. However, while the two former scores are mostly used at entry to risk-stratify patients by severity of illness, the latter also applies to quantify evolution of the patient's severity of illness and even benchmark intensive care unit performance [4]. Furthermore, beyond reporting a better record of the course of the disease, it allows for an evaluation of the impact of new treatments on patient outcome [5].

However, to evaluate whether treatment administration influences the course of organ failure, statistical analysis is often based on naive comparisons across randomized groups over time [6-8]. Mixed-effects models, which incorporate repeated measurements of SOFA over time in the same patients, appear to be a well established method for studying the relationship between treatment and the SOFA course. However, given the strong association between organ dysfunction and mortality for critically ill patients, the occurrence of death could result in non-trivial missing data for the longitudinal process. This is likely to provide biased results [9-12].

In a setting where the longitudinal observations may be correlated with survival, joint models of longitudinal and survival processes have been increasingly proposed in the past decade to recover information from these potentially informative censorings [10-26]. Mostly, a Gaussian mixed-effects linear sub-model is assumed for the longitudinal response, although a t-distribution which has a longer tail and thus is more robust to outliers, has been recently proposed [27], and a semi- or fully-parametric survival sub-model fits the survival times. Association between both longitudinal response and survival time is modeled through a zero-mean latent random process, and given all of the random effects, longitudinal measurements and survival times can then be assumed to be conditionally independent.

However, most joint models developed thus far in the literature have focused on univariate time-to-event data, where right censoring of the data acts independently of the survival process under study. In contrast, in the ICU setting, patients discharged alive are likely to be informatively censored. Thus, the analysis of survival data in the ICU in the setting of competing risks has been recently proposed to offer significant advantages over standard survival analyses [28,29]. Notably, they allow taking the time dependency of risk factors and competing events into account [30].

To study the effects of a covariate in competing risk settings, Cox analysis of cause-specific hazards has long been the technique of choice. Thus, the joint modeling of longitudinal and competing risk data that has been increasingly studied for the past four years first employed the cause-specific hazard sub-model, with a separate latent association between longitudinal measurements and each cause of failure [21,27,31-34]. However, although proportional cause-specific hazards modelling is the standard regression model of choice to handle competing risks, results may be difficult to interpret in terms of the cumulative event probabilities. Many authors have noted that the effect of a covariate on the cause-specific hazard function of a particular failure type may be very different from its effect on the cumulative incidence function [28,35-37]. For supporting clinical decision making, such cause-specific crude cumulative incidence, also known as the cause-specific subdistribution function, which is the probability of the occurrence of a specific event of interest, is widely recognized as clinically useful. This has led to the development of the proportional subdistribution hazards model [36], which offers a synthesis of single cause-specific hazards analyses.

In this paper, we propose a joint random effects model for a longitudinal marker and competing risks data that comprises a proportional subdistribution hazards submodel for the competing risks failure time data. We use the Markov chain Monte Carlo technique of Gibbs sampling to estimate the joint posterior distribution of the unknown parameters of the model, as previously proposed [14,31,38]. The paper is organized as follows. First, the ICU data is briefly presented. The next section describes the statistical joint model for the longitudinal and dropout processes.

The performance of our method is evaluated and compared with the cause-specific hazards submodel using both simulated data and the ICU clinical trial. Finally, a discussion is provided in the last section.

## Methods

### Motivating example

We analyzed data from an ongoing double-blind, parallel-arm, randomized clinical trial, conducted with 1,401 critically ill patients. Since our analyses only aim at illustrating modeling approaches of ICU data and due to the blind allocation of treatment arms, they will be referred as arm A (n= 703) and arm B (n= 698) hereafter. The median age was 63 (95%*CI*: 48-74) years, and 854 of the participants (61.0%) were men. At randomization (day 0), 664 (47.4%) patients presented with sepsis and 124 (8.9%) with trauma. Main characteristics were well balanced between randomized groups (Table 1).

**Table 1:** Main characteristics of patients according to randomized arm

N (%)		Arm A (n = 703)	Arm B (n = 698)	*p value*
Age (≥63 years)		360 (51.2)	347 (49.7)	0.59
Male Gender		423 (60.2)	431 (61.7)	0.54
Inclusion strata	Trauma patients	62 (8.8)	62 (8.9)	
	Sepsis patients	329 (46.8)	335 (48.0)	0.89
	Other patients	312 (44.4)	301 (43.1)	
SOFA score, median[Q1-Q3]		7.0 [5.0-7.75]	7.0 [5.0-7.74]	0.94

**Figure 1:**
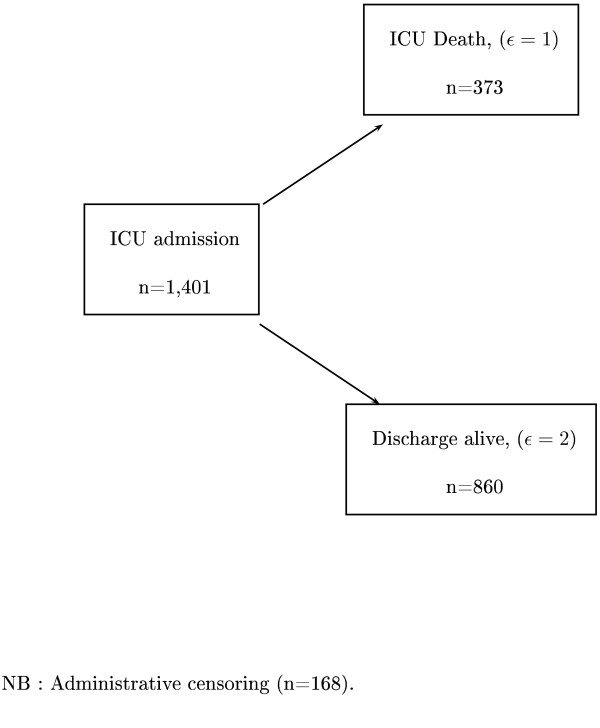
**ICU data: Graphical representation of the competing risk setting**.

All patients were followed until ICU discharge or day 28, whichever occurred first. The main endpoint was survival within 28 days following randomization. Of the 1,401 patients admitted to the ICU, 373 (26.6%) died in the ICU before day 28, 860 (61.4%) were discharged alive from the ICU within the first 28 days, and 168 (12.0%) were still alive in the ICU at day 28, thus administratively censored. Figure 1 summarizes the competing risk setting.

**Figure 2:**
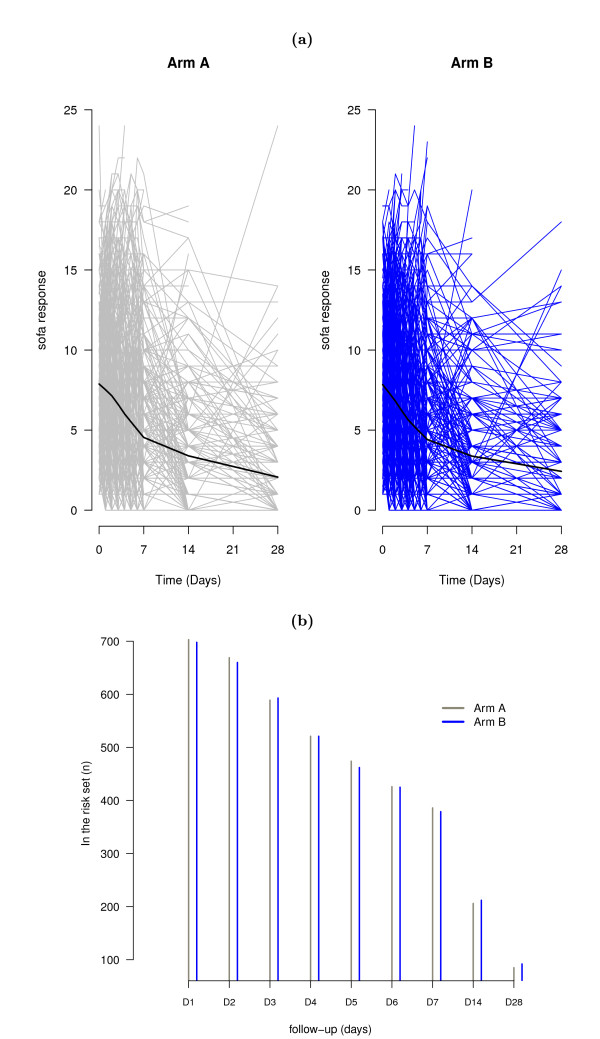
**Exploratory plots of longitudinal ICU data**. Exploratory plots of longitudinal ICU data: Evolution of individual SOFA scores with non parametric smooth curve (a) and risk sets/number of patients still alive and in the ICU (b), stratified by treatment group.

During the ICU stay, the SOFA score, which was measured every day up to day 7, then on day 14 and day 28 unless the ICU discharged the patient, was used to define a secondary endpoint of treatment effectiveness. We were thus interested in estimating the treatment effect on SOFA score, that is, on assessing whether its course over time could be considered as different in the two randomization arms or not (Figure 2a). As depicted by the non parametric smooth curve from trial data [39], a linear time average trend was considered. Indeed, the average SOFA decreases over time monotonically, and quadratic time trends appeared unlikely though possibly more rapidly within the first seven days. Of note, this could be explained by the data due to the absence of any time points after day 7 except at day 14 and day 28, with a marked decrease of information after day 7 (as plotted in Figure 2). Thus, we decided to only introduce a global linear time trend in the model. We also adjusted for potential confounders, namely age (dichotomized according to the sample median, 63 years), gender, and randomization strata (sepsis or other presentation mode). Age was analyzed as binary, though grouping may be seen as an extreme form of rounding with a resulting loss of information and power to detect real relationships [40]. However, continuous variable would require that the true risk increases (or decreases) monotonically with the level of the variable. Thus, we preferred to avoid such an additional assumption and to focus on the modeling of longitudinal response. Estimated coefficients and standard errors from the longitudinal model are displayed in Table 2. As expected, there was a significant decreasing pattern of the SOFA score over time, on average, by 0.22 points each day (95%*CrI*: -0.23, -0.20). However, the significance of the interaction term between the treatment group and time indicates that this developing trend of SOFA for the two treatment groups was different: indeed, during one day, the SOFA decrease for one group was 0.02 (95%*CrI*: -0.04, -0.003) less than that of the other group. The SOFA score course was also affected by the patient gender - with higher average SOFA values in males than in females - and the randomization strata - where the SOFA score values were on average higher in the case of septic patients.

**Table 2:** Separate modeling of SOFA course

Longitudinal	(Posterior mean (95% CI)	
Intercept	6.14	(5.66, 6.63) *
Time	-0.22	(-0.23, -0.20) *
Treatment group	0.24	(-0.16, 0.67)
Time × Treatment group	-0.02	(-0.04, -0.003)*
Age ≥ 63 years	0.53	(0.11, 0.94) *
Male Gender	0.46	(0.03, 0.88) *
Septic patients	1.45	(1.03, 1.86) *

Separate modeling of SOFA course - Posterior estimates of all fixed covariate effects in the longitudinal separate sub-model. * indicates a p-value < 0.05 based on t-tests for the individual fixed effects in the longitudinal model.

**Figure 3:**
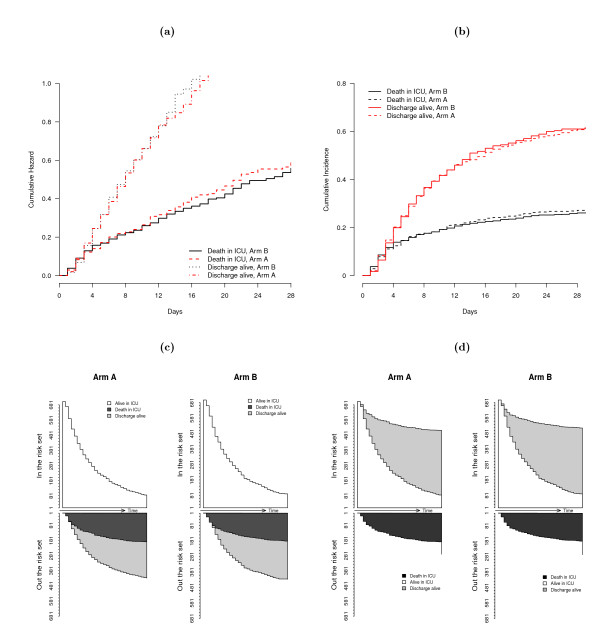
**Exploratory plots of survival competing risks ICU data**. Exploratory plots of survival competing risks ICU data, either based on cumulative cause-specific hazards stratified by treatment group (a) or cumulative incidence for discharge and death (b) stratified by treatment group; Respective risk sets/number of patients still alive and in the ICU stratified by treatment group are displayed in figures c and d, respectively.

However, once a patient is discharged from the ICU either alive or dead, he (she) drops out of the study, and hence, no longitudinal measures of the SOFA scores can be collected thereafter. This explains why some trajectories from Figure 2a are shortened as compared to the others, resulting in risk sets of exposed individuals (still alive in ICU) that decrease as time passes (Figure 2b). Thus, some selection bias is possibly introduced since analyzed populations over time differ from that originally enrolled in the trial. Indeed, since SOFA scores are time-dependant covariates that impact the outcome, either death or discharge alive, it is likely that deaths and discharges from the ICU should be considered as informative dropouts of the longitudinal SOFA process. To represent the occurrence of informative dropouts, two main approaches have been developed in the setting of competing risks, that differ in terms of the underlying function of interest and thus, risk sets. The cause-specific cumulative hazard of the Cox model displays the cumulative risk of failure from each cause of failure, conditionally on being free of any cause of failure (Figure 3a). Thus patients considered at risk of death in ICU at time *t *are only those who were not discharged alive before *t *(Figure 3c). By contrast, the subdistribution hazard could be obtained directly from the cumulative incidence function of failure (Figure 3b), where patients having been discharged alive before *t *are considered at risk of death thereafter (Figure 3d). It is obvious from Figures 3c and 3d that the size of the risks set decreases more rapidly over time in the cause-specific hazard model, due to the disappearance of discharges alive. In the model for the subdistribution hazard, the risks sets are only affected by the occurrence of deaths in ICU. Both approaches may interfere with the resulting estimates on longitudinal response. Thus, to look for the information provided by these covariates on ICU deaths and live ICU discharges, we fitted two competing risks models, that of cause-specific hazards and that of Fine and Gray [41], both incorporating the same binary prognostic covariates. Estimates of cause-specific and sub-distribution hazard ratios are reported in Table 3. There was no evidence of any treatment effect on either failure cause. Regarding the prognostic factors of ICU death, old age was significantly selected as positively associated with both cause-specific and sub-distribution hazards. Indeed, elderly people had an increased death risk in the ICU and were less likely to be discharged alive from the ICU. Conversely, the cumulative incidence of live discharges from the ICU was reduced in the oldest patients. Otherwise, the decreased cause-specific hazard of live discharge from the ICU observed in septic patients significantly affected the cumulative incidence of ICU discharges. However, this did not translate into any significant increase in the cumulative incidence of ICU death in septic patients, due to their decreased cause-specific hazard risk of death.

**Table 3:** Posterior mean hazard ratio estimates from separate survival models for competing risk data

		Cause specific hazards		Subdistribution hazards	
ICU DEATHS	No deaths/No pts	HR	95%*CI*	SHR	95%*CI*
Treatment group					
					
A	191/703	1		1	
B	182/698	1.04	(0.85,1.27)	1.03	(0.85,1.26)
Age					
					
<63 years	151/717	1		1	
≥63 years	222/684	1.40	(1.14,1.73)*	1.58	(1.28,1.94)*
Gender					
					
Female	150/547	1		1	
Male	223/854	0.90	(0.73,1.10)	0.95	(0.78,1.16)
Entry mode					
					
Other	185/737	1		1	
Sepsis	188/664	0.95	(0.77,1.16)	1.09	(0.89,1.33)

DISCHARGE ALIVE	No events/No pts				

Treatment group					
					
A	429/698	1		1	
B	431/703	0.99	(0.87,1.13)	1.00	0.87,1.14)
Age					
					
<63 years	493/717	1		1	(0.59,0.77)*
≥63 years Gender	367/684	0.71	(0.62,0.81)*	0.67	
					
Female	330/547	1		1	
Male	530/854	0.92	(0.80,1.06)	0.95	(0.83,1.09)
Entry mode					
					
Other	486/737	1		1	
Sepsis	374/664	0.72	(0.63,0.83)*	0.76	(0.67,0.87)*

Posterior mean hazard ratio estimates from separate survival models for competing risk data, namely, the cause-specific hazard (HR) model and the sub-distribution hazard (SHR) model. * indicates a p-value < 0.05 based on Wald tests.

We questioned whether incorporating such prognostic information on dropouts would modify estimates of covariate effects on the SOFA course as exposed above.

### The joint model formulation and estimation

Our joint model consists of the two linked submodels, a linear mixed model and a competing risks model.

#### Longitudinal submodel

Suppose there are *n *patients in the study indexed by *i *= 1,2,...,*n*. Let *Y_ij _*be a measure of the *j^th ^*SOFA score for patient *i *at time *t_ij _*, and ***Z***_*i *_denote the *p*-vector of explanatory variables (including treatment arm) measured at time 0 in patient *i*. The longitudinal submodel consists of a linear model with random effects:(1)

where *β *= (*β*_0_, *β*_1_) is a parameter vector of regression coefficients commonly referred to as fixed effects in the model; *ε_ij _*~ *N*(0, σ^2^) denotes the zero-mean Gaussian measurement error: we assumed that *ε_im _*was independent of *ε_is _*for any *m *≠ *s*; *W*_1*i*_*(t_ij_) *refers to the subject-specific random effects, that is, the value at time *t_ij _*of an unobserved zero{mean Gaussian random process. Following previous reports [33,42], random slope and random-intercept and -slope models were considered, namely *W*_1_*(t) *= *U*_1_*t *or *W*_1_*(t) *= *U*_0 _+ *U*_1_*t*, where (*U*_0_, *U*_1_) are zero-mean bivariate Gaussian variables.

#### Competing risks submodel

Let *T_i _*denote the failure time of patient *i*, and *k_i _*be the cause of failure from two possible causes, where *k_i _*= 1 denotes an ICU death and *k_i _= *2 denotes a live discharge from the ICU. Let *C_i _*denote the non informative censoring time. Let δ*_i _*= {*I*[*T_i _*≤ *C_i_*] × *k_i_*} be the event indicator, where δ*_i _*= *k_i _*in case of failure and δ*_i _*= 0 for non-informative censoring.

The submodel (3) specifies the distribution of the competing risks survival data. It is an extension of the subdistribution hazard model for competing risks survival data described by Fine and Gray [36](2)

in which *λ_0,k_(t) *is a non specified baseline subdistribution hazard for failure type *k*. It appears as a model analogous to the Cox model but based on subdistribution hazards, which is also known as the hazard associated with the crude cumulative incidence function, widely recognized as clinically useful for supporting clinical decision-making [43,44].

To model the correlation between different failure types, the Fine and Gray model was thus extended by incorporating a second zero-mean latent Gaussian process, :(3)

where  represent the fixed effects of **Z **on the two competing risks, *k *= 1, 2, respectively.

#### Submodel links

Failure times were associated with the longitudinal response through the latent Gaussian processes *W*_1_*(t)*, and , that were assumed to be proportional, i.e.: , where the parameters γ^(*k*) ^indicate the level of association between the two components of the joint model. Of note, positive values of γ^(*k*) ^suggests that positive values for associated random effects increase the hazard of "failure", while negative values of γ^(*k*) ^suggest that the positive values for the random effects decrease the chance of experiencing the event of interest. We further assumed that *W*_1 _and  were independent of the measurement errors *ε_ij_*. At last, the longitudinal measurements and competing risks survival times were assumed to be conditionally independent, given the covariates and random effects.

#### MCMC sampling procedure

The standard likelihood approach to this problem involves integration of the two sub-models over the distribution of random effects, which requires numerical integration since the two models are not conjugate. As an alternative, to estimate the parameters of interest, we used the Markov-chain Monte-Carlo method of Gibbs sampling to generate the posterior distribution of all unknown parameters of the joint model, given only the observed data.

**Figure 4:**
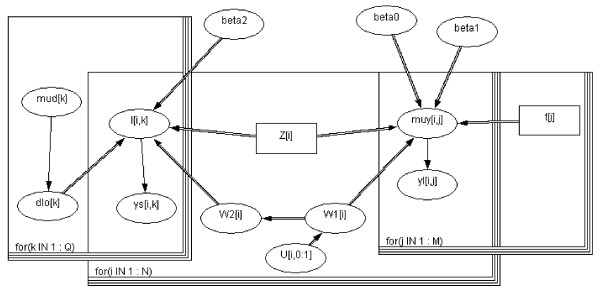
**Winbugs simplified Directed Acyclic Graph (DAG) for the joint model**.

Such models can be first represented by a *DAG *(Directed Acyclic Graph) (figure 4), which is the graphical representation of the structural model assumptions. The structural model specifies all observables variables and all unobservable parameters and how these quantities are related. Model quantities are represented by nodes in the graph: stochastic nodes for random quantities, logical nodes for functions of other parameters, and constant nodes for fixed quantities. We used non-informative priors for all parameters. The stochastic parameters (*β*(*s*) are given proper but minimally informative prior distributions, while the logical expression for precision (σ(*s*)) allows the standard deviation of the random effect distribution to be estimated. The fixed regression coefficients *β*_1 _and *β*_2 _were assigned a vague Gaussian prior. The initial values of the parameters for sampling were obtained by modeling the longitudinal data and survival data separately. The method involves iteratively sampling over a large number of cycles from the full conditional distributions of each parameter given the current assignment of all other parameters and data. After discarding the early samples to allow the process to converge, we used subsequent realizations of each parameter for summarizing the posterior distribution. We used the posterior means and variances of the Gibbs samples to describe the results and used the 2:5*^th ^*and 97:5*^th ^*percentiles of the empirical distributions to estimate the 95-percent credible intervals (95%*CrI*). To assess convergence, we used the guidelines proposed by Spiegelhalter [45]. In the analyses, the results were based on three parallel MCMC sampling chains of 30,000 iterations each, after a burn-in of 1,000 iterations. From model alternatives of linked structures, we choose the most parsimonious model according to the Deviance Information Criterion (DIC) [46]. Joint models were fitted through the use of the WinBUG_S _package [45] and the R package 'BRugs'. Separate models were fitted using standard statistical packages (R package cmprsk with 'cuminc' function for cumulative incidences, the R package nlme for longitudinal analyses, and WinBUGs) though other statistical packages such as SAS (SAS Inc., Cary, NC) could have been used [47].

## Results and discussion

### Application revisited

Table 4 displays posterior summary statistics for the regression coefficients related to the SOFA score course together with the dropout processes. Regardless of the survival sub-model used for competing risks, the best fitted model links the sub-models through a linear combination of random effects; that is, we use the intercept- and slope- random effect model *W*_1 _= *U*_0 _+ *U*_1_*t *(based on the DIC value). The marginal assumption of normality of random effects appeared to hold reasonably, on the basis of the residual plots (data not shown). Expectedly, a negative association between the two model components was observed regardless of the competing risk sub-model, with γ^(1) ^estimated at about 3 and γ^(2) ^at about -3. Indeed, this could have been anticipated, since deaths are more likely to occur in patients with high SOFA values, whereas patients with high SOFA scores are less likely to be discharged alive from the ICU. Previous estimated treatment effects on the mean SOFA course and dropouts were affected by the model assuming ICU deaths and live discharges from the ICU as informative censoring observations. Besides the prognostic value on the SOFA score of gender, age and sepsis, which remained regardless of the joint model used, the treatment effect on either outcome was interestingly modified by incorporating a correlation between the SOFA course and dropouts. The modeling of the dropout process using cause-specific hazards erased the time by treatment interaction on the SOFA course, while that based on sub-distribution hazards did not. However, interestingly, estimated treatment effect on the hazards of death increased whatever the survival sub-model, either for cause-specific or sub-distribution hazards, but only reached statistical significance with the later. Similarly, in the survival sub-models, the separate and joint analyses produced differences in estimates for other parameters such as male gender and sepsis. Actually, all these differences between the separate and joint analyses might be explained by the negative significance for the covariance between the latent variable of the longitudinal model and that of the survival model.

**Table 4:** Posterior estimates for the ICU data based on joint models

Modeling of dropouts	Cause specific hazards		Subdistribution hazards	
**Sub-model**				

**LONGITUDINAL**	**Posterior mean**	**(95% *CrI*))**	**Posterior mean**	**(95% *CrI*))**
				
Intercept	6.29	(6.05, 6.53)*	6.24	(5.74, 6.75)*
Time	-0.05	(-0.07, -0.04)*	-0.06	(-0.07, -0.05)*
Treatment group	0.34	(0.10, 0.58)*	0.26	(-0.17, 0.68)
Time × Treatment group	-0.012	(-0.04, 0.01)	-0.02	(-0.03, -0.003)*
Age (years)	0.45	(0.27, 0.63)*	0.53	(0.12, 0.96)*
Male Gender	0.44	(0.25, 0.63)*	0.47	(0.02, 0.96)*
Septic patients	1.33	(1.15, 1.51)*	1.44	(1.01, 1.86)*
				

SURVIVAL	HR	(95% *CrI*))	SHR	(95% *CrI*))
				
DEATHS				
				
Treatment group	1.22	(0.98, 1.53)	1.23	(1.00, 1.53)*
Age (years)	1.93	(1.55, 2.41)*	1.38	(1.10, 1.74)*
Male Gender	1.19	(0.96, 1.50)	1.35	(1.07, 1.69)*
Septic patients	1.38	(1.11, 1.72)*	1.18	(0.95, 1.46)
				
γ^(1)^	3.32	(3.09, 3.57)	3.42	(3.24, 3.61)

DISCHARGES				
				
Treatment group	1.17	(1.02, 1.36)*	1.25	(1.08, 1.43)*
Age (years)	0.91	(0.79, 1.05)	1.08	(0.93, 1.24)
Male Gender	1.25	(1.07, 1.45)*	1.30	(1.11, 1.51)*
Septic patients	1.02	(0.88, 1.18)	1.12	(0.96, 1.29)
				
γ^(2)^	-2.96	(-3.11, -2.80)	-3.35	(3.48, -3.22)

The upper table displays longitudinal joint estimates, and the lower table displays the survival estimates in cause-specific hazards and sub-distribution hazards. * indicates statistically significance at level 0.05 (in the Bayesian sense; 95% credible set excludes 0)

### A Simulation study

#### Sampling details

In this section, we conducted a simulation study to illustrate the method, to examine the feasibility as well as properties of the proposed joint model. We simulated the complete data from the following intercept- and slope- random model.

Longitudinal data were generated for *n *subjects from the Gaussian linear model as given by (4). The change in the subject's longitudinal biomarker over time is described using a linear mixed-effects model in which the subject-specific effects are captured by latent variables. We consider binary covariate, continuous covariate (time covariate), and an interaction between binary and time covariate along with the usual intercept term. Thus, a random intercept-and-slope model is adopted, such that *W*_1_(*t*) = *U*_0 _+ *U*_1_*t*:(4)

where *t_ij _*= 0, 1,....,7, 14, 28 represent the visit scheduled times, *X_i _Bernoulli*(0:5) acts as the treatment indicator in a randomized clinical trial. The random intercept *U*_0*i *_and slope *U*_1*i *_were assumed normally distributed as *N*(0, σ*U*_0 _) and *N*(0, σ*U*_1 _), respectively, and independent of the measurement error *ε_ij_~N*(0, 0.1). Estimates from separate analysis of the longitudinal and the time to event components were reasonable starting values for the model.

For simplicity, we considered only two competing risks (*k *= 2), with failure times data generated through the method described by Fine and Gray [41] but including frailty. Distinct baseline hazards were used for each risk, and the same binary covariate as used in the longitudinal sub-model was incorporated into the competing-risks sub-models. Briefly, the sub-distribution for the failures of interest are given by:(5)

which is a unit exponential mixture with mass 1 - *p *at ∞ when *X *= 0, and uses the proportional subdistribution hazards model to obtain the subdistribution for nonzero covariate values. The subdistribution for the competing risks failure cause was then obtained using an exponential distribution with rate exp . As detailed above, proportional association between the longitudinal data and the competing risks was generated by setting .

Censoring times were generated from an exponential distribution with rate 0.25. We used the true parameter value of *p *= 0.25, for *n *= 100, 500 subjects. This gave 25 per cent cause 1 failures, 65 per cent cause 2 failures, and 10 per cent of censoring.

Based on the collection of previous analyses illustrated in Tables 2 and 3, the following proper priors were used for *β*_10_, *β*_11_, *β*_12_, *β*_13_, and . The method used informative priors for some parameters with the prior means (*β's*) set as the true parameter values. Setting γ = 1 induces a positive association between the competing risks. We also set γ = -1 to induce negatively associated competing risks, that may apply when discharge is a competing cause of failure, as observed in the motivating exemple. Longitudinal responses were missing after the observed or censored event times, with an averaged number of total longitudinal observations of 7:0 per subject. This is consistent with the example findings (6:6 observations per subject). After a burn-in phase of 1,000 iterations, eliminated from the sample to avoid influence of initial parameters, we used means and standard deviations of a single series of 10,000 Gibbs samples as point estimates of the parameters and their standard errors. A total of 100 simulations were performed. Simulations were carried out in R language (R Development Core Team) [48].

#### Simulation results

Estimated bias and standard deviations (SD) of the posterior means are reported in Table 5. First of all, the intercept *β*_10 _and time trend *β*_11 _of the longitudinal measurements were overestimated when the sample size is not that large (*n *= 100). Our results are consistent with the findings of Elashoff et al. [21], who reported that biases and/or variances for these parameters were larger for the joint model than the separate. However, smaller biases and variances could be observed with larger sample sizes, and we are able to obtain almost unbiased estimates for all the quantities in the joint analysis based on 500 subjects. Accurate estimation of the association parameters γ was obtained. At last, we note that the joint model could be conservative in the sense that the estimated standard errors tend to be slightly larger than the true ones.

**Table 5:** Simulation study

	Positive association		Negative association	
		
Parameter	True value	Bias (SD)	True value	Bias (SD)
n = 100				
				
*Longitudinal*				
Intercept *β*_10_	6.15	0.24 (0.16)	6.15	0.20 (0.16)
Time *β*_11_	-0.25	0.22 (0.03)	-0.25	0.19 (0.04)
Binary covariate *β*_12_	0.25	0.01 (0.19)	0.25	0.01 (0.20)
Interaction Time × Binary covariate *β*_13_	0.0	-0.03 (0.03)	0.0	-0.05 (0.03)
				
*Survival*				
Binary covariate *β*_2_	0.0	0.25 (0.34)	0.0	0.39 (0.35)
γ	1.0	-0.09 (0.12)	-1.0	0.13 (0.12)
*Variances*				
σ_*U*0_	1.0	0.04 (0.14)	1.0	0.02 (0.13)
σ_*U*1_	1.0	0.02 (0.13)	1.0	0.02 (0.13)

n = 500				
				
*Longitudinal*				
Intercept *β*_10_	6.15	0.20 (0.16)	6.15	0.23 (0.04)
Time *β*_11_	-0.25	0.02 (0.02)	-0.25	0.05 (0.05)
Binary covariate *β*_12_	0.25	0.04 (0.22)	0.25	0.03 (0.06)
Interaction Time × Binary covariate *β*_13_	0.0	-0.007 (0.04)	0.0	-0.01 (0.01)
				
*Survival*				
Binary covariate *β*_2_	0.0	0.12 (0.29)	0.0	-0.16 (0.12)
γ	1.0	-0.03 (0.09)	-1.0	-0.04 (0.10)
				
*Variances*				
σ_*U*0_	1.0	0.02 (0.14)	1.0	0.04 (0.12)
σ_*U1*_	1.0	0.03 (0.14)	1.0	0.03 (0.13)

Simulation study, bias and standard deviations (SD) estimates of the posterior means for competing risk joint model, sample size = 100, 500.

## Discussion

In this paper, we aimed at comparing two treatment groups with respect to the course of the SOFA score in critically ill patients. Analysis was complicated by informative dropouts, since once a patient has been discharged, either alive or dead, from the ICU, no longitudinal measure of the severity score of interest can be collected thereafter. Thus, when analyzing such data, separate modeling of the SOFA score, that is, ignoring the dropout process, is likely to be inappropriate and one should obtain less biased and more efficient inferences using joint models Actually, joint models allow incorporating informative censoring and time by treatment interaction, and provide complementary information when assessing how the treatment manifests itself through the marker [49].

Such joint models in this particular setting required modelling assumptions. First, to assess time by treatment interaction on the SOFA score, a linear time effect was assumed. Indeed, as depicted in Figure 2a, the average SOFA monotonically decreases over time, so that quadratic time pattern was unlikely. Of note, the observed profiles may have suggested a change point at 7 days in the slope whatever the treatment arm, so that a piecewise mixed-effects model could have been fitted. However, we only introduced one linear time trend because of the data, due to the absence of any time points after day 7 except at day 14 and day 28, and due to the increased amount of informative dropouts over time, notably after day 7 (Figure 2b). Secondly, in the particular setting of ICU data, where dropouts result from either deaths or live discharges, models for competing risk failure time data should be used to fit the survival responses [28-30]. Since the SOFA score was actually measured only in patients during their ICU stay, the possibly informative dropout process of interest was clearly that of ICU deaths/discharges. This avoided open issues with regards to the primary outcome to be used in the ICU, as well as on the model to be fitted to such data -either based on binary [50] or survival data analysis techniques [30]. Joint models could also apply to other competing risks settings such as those published by the UCLA team in scleroderma-related interstitial lung disease with intermittent measures of forced vital capacity which were informatively censored by study withdrawal due to disease or treatment related reasons [27,32,51]. Based on our simulation study, we observed that an increase in the sample size decreased the estimation bias for the parameters in both submodels. However, we observed, as also noted by Hu [38] in their own model, that the implemented method is sensitive to outliers. A further development will be to implement a more robust joint model for longitudinal and survival data.

A few authors have already proposed a joint modeling of longitudinal and multivariate survival data [31,33,38]. Our proposed approach differs from those in two main points. First, a cause-specific hazard sub-model [33] or a frailty model [31] has been conventionally used to handle several types of failures; we decided instead to fit to a sub-distribution hazard sub-model [36] to provide estimates of treatment effect directly related to the cumulative incidence of dropouts [43,44]. Secondly, an EM algorithm was used for inference purposes in [33]. Actually, the Bayesian framework of Chi in 2006 [31] motivated our investigation of a Bayesian alternative that allows full and exact posterior inference for any parameter or predictive quantity of interest. Thus, we developed a fully Bayesian approach, implemented via MCMC methods using WinBUGS software, as previously reported [14,31,52,53]. Such a Bayesian method for joint modeling of longitudinal and competing risks survival data was very recently reported in the setting of cause-specific hazards sub-model [38]. We illustrated in this paper how the joint model strategy may affect the results. Our results suggest that the treatment effect on the SOFA course in separate modeling of the SOFA course could be evidenced when considering informative censoring modeled by sub-distribution hazards. The significant treatment by time interaction was erased by the modeling of informative dropouts throughout cause-specific hazards.

In the setting of joint modeling of competing risks together with that of longitudinal response, such a difference in the handling of competing risk outcomes based on the Fine and Gray model appears to translate into the observed difference in treatment effect on the longitudinal outcome. This makes clear the requirement for statistical analysis of such data to be clearly planned in the protocol of such studies. Other approaches, such as marginal structural models for non-dynamic treatment regimes, appear to be of prime interest in this setting [54,55].

Valid inference requires a framework in which potential underlying relationships between the event and longitudinal process are explicitly acknowledged. Latent variable models used in this context do not directly model the association between longitudinal and survival response, but rather focus on correlated, latent random effects. The random intercept-and-slope model was found to give a significantly improved fit (by DIC) above other models examined such as the random intercept-only model. This is similar to that reported by Williamson [33]. Additionally, Henderson *et al. *[15] described a latent variable association model, and Lin *et al. *[56] concentrated on latent class mixed models. In a recent paper, Liu [57] showed that the hazard of death may be dependent on random effects from various levels. In this way, Tsiatis and Davidian provided a nice overview of joint models [12], describing further details on underlying assumptions statements and on the likelihood of model parameters in such models.

## Conclusions

The consideration of joint models permits useful analysis of very complex data. It could help to improve estimation of the impact of proposed prognostic features on the main endpoints in the trial. We proposed a method that gives accurate estimates, and a Bayesian alternative that permits full and exact posterior inference for any parameter or predictive quantity of interest.

## Competing interests

The authors declare that they have no competing interests.

## Authors' contributions

It was SC's idea to introduce the subdistribution hazards submodel for competing risks data in the joint modeling of longitudinal data and of survival data in the presence of informative censoring. ED conducted the statistical analysis. Both prepared and finalized the manuscript.

## Pre-publication history

The pre-publication history for this paper can be accessed here:

http://www.biomedcentral.com/1471-2288/10/69/prepub
